# Association of Nitric Oxide Synthase and Matrix Metalloprotease Single Nucleotide Polymorphisms with Preeclampsia and Its Complications

**DOI:** 10.1371/journal.pone.0136693

**Published:** 2015-08-28

**Authors:** Daniela P. Leonardo, Dulcinéia M. Albuquerque, Carolina Lanaro, Letícia C. Baptista, José G. Cecatti, Fernanda G. Surita, Mary A. Parpinelli, Fernando F. Costa, Carla F. Franco-Penteado, Kleber Y. Fertrin, Maria Laura Costa

**Affiliations:** 1 Hematology and Hemotherapy Center, University of Campinas—UNICAMP, Campinas, São Paulo, Brazil; 2 Department of Obstetrics and Gynaecology, School of Medicine, University of Campinas—UNICAMP, Campinas, São Paulo, Brazil; 3 Department of Clinical Pathology, School of Medicine, University of Campinas–UNICAMP, Campinas, São Paulo, Brazil; University Francisco de Vitoria School of Medicine, SPAIN

## Abstract

**Background:**

Preeclampsia is one of the leading causes of maternal and neonatal morbidity and mortality in the world, but its appearance is still unpredictable and its pathophysiology has not been entirely elucidated. Genetic studies have associated single nucleotide polymorphisms in genes encoding nitric oxide synthase and matrix metalloproteases with preeclampsia, but the results are largely inconclusive across different populations.

**Objectives:**

To investigate the association of single nucleotide polymorphisms (SNPs) in *NOS3* (G894T, T-786C, and a variable number of tandem repetitions VNTR in intron 4), *MMP2* (C-1306T), and *MMP9* (C-1562T) genes with preeclampsia in patients from Southeastern Brazil.

**Methods:**

This prospective case-control study enrolled 77 women with preeclampsia and 266 control pregnant women. Clinical data were collected to assess risk factors and the presence of severe complications, such as eclampsia and HELLP (hemolysis, elevated liver enzymes, and low platelets) syndrome.

**Results:**

We found a significant association between the single nucleotide polymorphism *NOS3* T-786C and preeclampsia, independently from age, height, weight, or the other SNPs studied, and no association was found with the other polymorphisms. Age and history of preeclampsia were also identified as risk factors. The presence of at least one polymorphic allele for *NOS3* T-786C was also associated with the occurrence of eclampsia or HELLP syndrome among preeclamptic women.

**Conclusions:**

Our data support that the *NOS3* T-786C SNP is associated with preeclampsia and the severity of its complications.

## Introduction

Preeclampsia is a multisystem pregnancy-specific disease, with varying clinical features, that affects 2–8% of pregnancies worldwide and is a major cause of maternal morbidity and mortality[1,2], especially in low and middle income countries[3,4], accounting for short and long-term health consequences for mothers and offspring[2,5]. There is a global concern on its rising incidence, mostly due to increasing rates of underlying and predisposing disorders such as chronic hypertension, diabetes and obesity[1,6].

The definition of preeclampsia was recently revised[7] to take into consideration new onset hypertension developing after 20 weeks gestation as well as the coexistence of one or more of the following: proteinuria, other maternal organ dysfunction (renal insufficiency, liver involvement; neurological complications or hematological complications) and/or uteroplacental dysfunction reflected by fetal growth restriction. Severe preeclampsia is considered in the presence of extremely high blood pressure (systolic blood pressure over 160mmHg and diastolic over 110mmHg), symptoms of impeding eclampsia, or diagnosis of HELLP (hemolysis, elevated liver enzymes, and low platelets) syndrome. According to time of onset of preeclampsia, early-onset is characterized when the disease presents before 34 weeks of gestation.

Despite extensive research on this topic, the exact causes of this malady remain largely unknown, with strong hypotheses implicating a disturbance of the placental function early in pregnancy that is associated with impaired spiral arteries remodeling and exacerbated inflammatory response[8]. The role of genetics is still not well defined; however, there is clear evidence of the risk factor played by family history, supported by twin studies, segregation and linkage analyses, as well as genome-wide association studies[9–12].

Several studies have identified single nucleotide polymorphisms (SNPs) that may be associated with preeclampsia. The *NOS3* gene is located on the long arm of chromosome 7 (7q35-q36 region) and encodes the enzyme endothelial nitric oxide synthase (eNOS)[13], responsible for the production of nitric oxide (NO). NO is involved in the regulation of blood pressure, blood flow distribution to the organs, inhibition of platelet adhesion and aggregation, and inhibition of some leukocyte functions[14]. Some SNPs including T-786C, G894T (Glu298Asp), and a variable number of tandem repeats (VNTR) in intron 4 have been identified[15–17] and can influence NO production.

The G894T polymorphism has been associated with a reduction in endothelium-dependent vasodilation in pregnancy and with the risk for ischemic heart disease[18,19]. However, in preeclampsia, the results have been conflicting: some studies show association of this polymorphism with the disease, while others disagree[20–25].

The T-786C SNP causes significant reduction of eNOS activity, may be associated with a predisposition to hypertension in general, and is also associated with reduced eNOS mRNA in the placenta[17,26]. One study has suggested the need for replication of their finding of this polymorphism as a risk factor for preeclampsia[24].

Similarly to eNOS, extracellular matrix metalloproteases (MMPs) are another group of enzymes that can modulate blood flow in the vessels. MMPs induce cleavage of calcitonin gene-related peptide, and of "big endothelin" (ET-1), producing its most active form, ET-1 [1–32], favoring vasoconstriction[27,28]. Increased activity of MMPs seems to play a role in preeclampsia, as plasma levels of MMP-2 have been found to be elevated in patients with preeclampsia[29], so SNPs in genes encoding MMPs have been studied as biomarkers of susceptibility to preeclampsia.

SNPs in the *MMP2* gene have been less studied than *NOS3*, and despite their association with increased levels of MMP-2, SNPs C-1306T and C-735T have not been identified as biomarkers of susceptibility in one population and warrant further studies[30].

SNP C-1562T in the gene encoding MMP-9 has been implicated in an increase in gene expression in individuals carrying the polymorphic allele T[31]. This SNP has been associated with hypertension in the general population[32–34] and with gestational hypertension, but susceptibility to preeclampsia has been controversial across different populations[30,35–38].

We aimed at shedding light on the controversial role of these polymorphisms by performing a case-control study of preeclamptic and normotensive pregnant women. We compared the distribution of genotypes for *NOS3* G894T, T-786C, and VNTR in intron 4, as well as *MMP2* C-1306T and *MMP9* C-1562T, and tested their association with preeclampsia and its major complications.

## Materials and Methods

### Ethics statement

This study was approved by the local Ethics Board of the Hospital da Mulher Prof. Dr. José Aristodemo Pinotti, Centro de Atenção Integral à Saúde da Mulher–CAISM, a public regional reference hospital for Obstetrics and Gynecology in Campinas, Brazil (approval number 502/2010). Written informed consent was obtained from all subjects.

### Patient enrollment

Inclusion criteria for cases were pregnancy between 20 and 40 weeks or post-partum, and preeclampsia as defined by new onset of hypertension with systolic blood pressure (BP) above 140mmHg or diastolic BP above 90mmHg, in association with either proteinuria above 300mg in 24h or a positive urine dipstick test equal to or above 2+ in the absence of urinary infection[39]. Inclusion criteria for controls were pregnancy between 20 and 40 weeks or post-partum and absence of any of the diagnostic criteria for preeclampsia until discharge after delivery, including a negative urine dipstick test or normal 24-hour proteinuria. Exclusion criteria for both groups were multiple gestation, fetal malformations, arterial hypertension, gestational hypertension, diabetes mellitus, autoimmune diseases, thyroid disease, and chronic infectious diseases.

### Genotype determination

Peripheral venous blood samples were collected in tubes containing EDTA and stored at 4 degrees Celsius until the time of processing. All samples were numbered upon enrollment and laboratory personnel were blinded to the diagnostic group allocation until the genotyping phase was finished. Genomic DNA was obtained using phenol-chloroform modified technique[40]. *NOS3* G894T and T-786C, *MMP2* C-1306T, and *MMP9* C-1562T genotyping were performed by PCR-RFLP analysis on a Veriti equipment (Applied Biosystems, CA., USA) followed by 3% agarose gel electrophoresis stained with ethidium bromide visualized under UV light. Table 1 summarizes primers, annealing temperatures, enzymes used, and fragments obtained. Genotypes for the VNTR of intron 4 of *NOS3* were determined by FAM-labeled PCR, subsequently diluted at a ratio of 1 to 10 with distilled water, added to a mixture containing formamide (Hi-Di, Applied Biosystems, CA, USA) and standard marker (LIZ 500, Life Technologies), to a final volume of 10uL. The amplified fragments were denaturated at 95°C and separated via capillary electrophoresis performed in an automatic DNA Analyzer (ABI 3500 Genetic Analyzer, Applied Biosystems, CA, USA). Automatic sequencing was performed for validation of PCR-RFLP using random samples including all genotypes in all three genes using the Big Dye Terminator Cycle Sequencing Ready Reaction Kit v3.1 (Applied Biosystems, CA, USA) according to the manufacturer's instructions for subsequent electrophoresis in automatic sequencer (ABI 3500 Genetic Analyzer). Sequences obtained were analyzed and compared with public databases (http://www.ncbi.nih.nlm.gov).

**Table 1:** Sequences of primers, annealing temperatures, and enzymes used in PCR-RFLPs and sizes of the fragments obtained.

SNP	Primer sequences	Fragment size (bp)	Annealing temperature	Restriction enzyme	Fragments in wild-type homozygotes (bp)	Fragments in polymorphic homozygotes (bp)
*MMP2* T-1306C	F-5´-CTTCCTAGGCTGGTCCTTACTGA-3´	193	58°C	*Xsp I*	186+7	162+24+7
	R-5´-CTGAGACCTGAAGAGCTAAAGAGC-3´					
*MMP9* C-1562T	F-5´-GCCTGGCACATAGTAGGCCC-3´	436	60°C	*Nla3*	381+48+7	246+135+48+7
	R-5´-CTTCCTAGCCAGCCGGCATC-3´					
*NOS3* G894T	F-5´-AAGGCAGGAGACAGTGGATGGA-3´	248	58°C	*MboI*	248	158+90
	R-CCCAGTCAATCCCTTTGGTGCTCA-3´					
*NOS3* T–786C	F-5´-CACCCAGGCCCACCCCAACT-3´	394	58°C	*Msp* I	394	352+42
	R-5´-GCCGCAGGTCGACAGAGAGACT-3´					
*NOS3* VNTR	F-5´-CTTACTCTCCACTGCTTTTCAGAG-3´ (Martines *et al*.)	394–448	58°C	NA	NA	NA
	R-5´-CGCAGGTCAGCAGAGAGACTAG-3´					

SNP, single nucleotide polymorphism; F, forward; R, reverse; bp, base pairs; VNTR, variable number of tandem repeats, NA, not applicable

### Statistical analysis

Clinical and laboratorial data were tabulated in a spreadsheet using Microsoft Excel 2003–2013 (Microsoft, USA) and subsequently exported and analyzed with GraphPad Prism 5.0 software (GraphPad Software, USA). Genotypic and association studies were performed using R software v3.1.2 (The R Foundation for Statistical Computing, USA) with the statistical analysis packages *SNPassoc* and *epicalc*.

## Results

A total of 383 pregnant women were enrolled in this study from March 2011 to July 2014. Forty samples were excluded due to inadequate storage of blood tubes compromising DNA quality or failure to collect minimum clinical data confirming case or control group allocation. There was a total of 77 cases of preeclampsia and 266 control pregnant women. Both groups did not differ significantly in height, pre-gestational weight, body mass index, the percentage of primigravidas, or of women reporting a smoking habit. Preeclamptic women presented at earlier gestational age for delivery, were more likely to undergo cesarean section, and their offspring was significantly less mature according to Capurro scores, with significantly lower birth weight (Table 2).

**Table 2:** Clinical characteristics of the study population.

	Controls (n = 266)	Cases (n = 77)	*P*
**Age,** y	24,5 (24,8–28,0)	26,4 (25,3–28,0)	0.0547
**Pre-gestational weight,** kg	64,0 (62,4–65,5)	66,3 (63,0–69,6)	0.2556
**Height**, m	1,60 (1,60–1,62)	1,61 (1,59–1,62)	0.4529
**Body mass index,** kg/m^2^	24,6 (24,1–25,1)	25,3 (24,0–26,6)	0.1613
**Gestational age at admission,** weeks	38,7 (38,4–39,0)	35,2 (34,4–36,0)	<0.0001
**Neonate weight,** g	3138 (3071–3205)	2304 (2082–2525)	<0.0001
**Neonate Capurro score,** weeks	38,5 (38,2–38,8)	35,3 (34,5–36,1)	<0.0001
**Smoking** (%)	25/258 (9.4)	7/74 (9.1)	0.4924
**Primigravida** (%)	117/266 (44.4)	38/77 (49.4)	0.1764
**Previous preeclampsia** (%)	15/265 (5.6)	28/77 (35.6)	<0.0001
**Type of delivery**			
Vaginal	157	13	
Forceps	11	1	<0.0001
Cesarean section	87	61	

Tables 3 and 4 present the data on genotypes according to SNPs and to group allocation, major allele frequencies and Hardy-Weinberg equilibria. We detected a higher frequency of the presence of SNPs in the *NOS3* gene in the group of pregnant women with preeclampsia, but not in the *MMP2* and *MMP9* genes. In regard to the VNTR in intron 4 of the eNOS encoding gene, we noticed a greater diversity of alleles in the control population of pregnant women, with a more frequent appearance of the rare alleles bearing 4 and 6 repetitions (Table 5). Since in all polymorphisms the alleles were in Hardy-Weinberg equilibrium, we assumed that our population had no significant inbreeding or specific migration issues that could affect our estimation of genetic frequencies.

**Table 3:** Major allele frequencies and Hardy-Weinberg tests for the study population.

SNP	MAF	HWE *P*
*NOS3* G894T	0.861	0.65
*NOS3* T-786C	0.907	0.51
*MMP2* C-1306T	0.747	0.77
*MMP9* C-1562T	0.705	0.60
*NOS3* VNTR in intron 4	0.805	0.59

SNP, single nucleotide polymorphism; MAF, major allele frequency; HWE, Hardy-Weinberg equilibrium

**Table 4:** Genotype frequencies of SNPs for the study population.

	Wild-type homozygote	Heterozygote	Polymorphic homozygote	Not determined
***NOS3894* genotype**	**GG**	**TG**	**TT**	**ND**
Cases	42 (55%)	25 (32%)	10 (13%)	0 (0%)
Controls	149 (56%)	101 (38%)	13 (3%)	3 (1%)
***NOS3–*786 genotype**	**TT**	**CT**	**CC**	**ND**
Cases	36 (47%)	29 (38%)	12 (16%)	0 (0%)
Controls	131 (49%)	118 (44%)	15 (6%)	2 (1%)
***MMP2–*1306 genotype**	**CC**	**CT**	**TT**	**ND**
Cases	53 (69%)	23 (30%)	1 (1%)	0 (0%)
Controls	196 (74%)	61 (23%)	4 (2%)	5 (2%)
***MMP 9–*1562 genotype**	**CC**	**CT**	**TT**	**ND**
Cases	60 (78%)	11 (14%)	1 (1%)	5 (6%)
Controls	217 (82%)	43 (16%)	3 (1%)	3 (1%)

ND, not determined

**Table 5:** Genotype frequencies of NOS3 VNTR in intron 4 for the study population.

*NOS3* VNTR intron 4 genotype	4/4	5/5	4/5	4/6	5/6	ND
Cases	0 (0%)	52 (68%)	17 (22%)	1 (1%)	0 (0%)	7 (9%)
Controls	13 (5%)	148 (56%)	78 (29%)	2 (1%)	4 (2%)	21 (8%)

VNTR, variable number of tandem repetitions; ND, not determined

Table 6 shows the results of univariate analysis for risk factors for preeclampsia. The most significant clinical risk factor was previous preeclampsia, while amongst genetic risk factors, only SNPs in the *NOS3* gene were associated with preeclampsia. Subsequent multivariate analysis showed that only *NOS3* T-786C was associated with preeclampsia independently from age, height, or weight.

**Table 6:** *Odds ratios* for risk factors for preeclampsia.

Risk factor	Odds ratio	95% CI
**Age (years)**	**1.04**	**1.01.-1.08**
Height (cm)	0.98	0.94–1.02
Weight (kg)	1.00	1.00–1.00
Smoking	0.96	0.33–2.40
**Previous preeclampsia**	**9.21**	**3.22–28.26**
*MMP2* C-1306T	0.85	0.09–7.68
*MMP9* C-1562T	1.22	0.13–11.91
***NOS3* G894T (recessive model, TT vs. GT+GG)**	**2.87**	**1.21–6.83**
***NOS3* T–786C (recessive model, CC vs. TC+TT)**	**3.06**	**1.37–6.87**

CI, confidence interval; values in bold were statistically significant.

**Fig 1:**
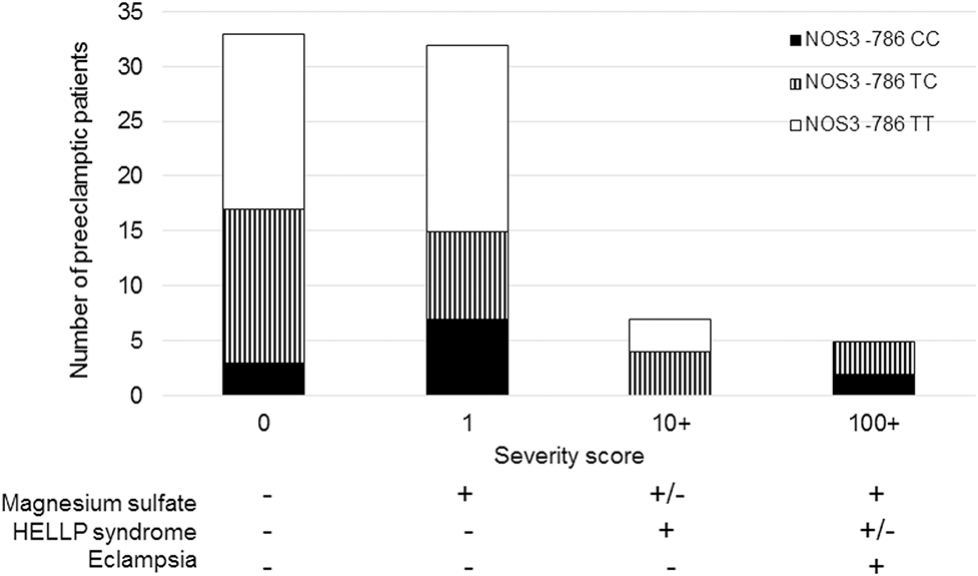
Number of preeclamptic patients according to severity score and genotype distributions for *NOS3* T-786C.

Among preeclamptic women, we also obtained clinical data whether they had to be admitted to receive magnesium sulfate to treat severe preeclampsia, if they developed HELLP syndrome, or eclampsia. Patients were then assigned a severity score according to the complications reported: baseline score for preeclampsia was zero, adding 1 point if admission to administer magnesium sulfate was indicated, 10 points if HELLP syndrome was diagnosed, and 100 points if eclampsia ensued, resulting in a score ranging from 0 to a maximum 111 points. We analyzed whether the *NOS3* T-786C polymorphism was associated with more severe preeclampsia among the cases. Simple severity score analysis showed that wild-type homozygotes TT for this SNP had a mean score of 1.39, while women with either a CT or CC genotype had a mean score of 14.15. A score above 10 meant that the latter genotype was possibly associated with at least HELLP syndrome. Complementary chi-square analysis of the subgroup of preeclamptic women showed that, in a recessive model, *NOS3* T-786C was significantly associated with the occurrence of either HELLP syndrome or eclampsia (*P*<0.0001). (Fig 1)

## Discussion

In this study, we have explored some clinical and genetic risk factors for preeclampsia in a Brazilian population. We have confirmed that preeclampsia represents a relevant maternal and neonatal comorbidity, with a lower median gestational age upon admission for delivery in women diagnosed with preeclampsia, as well as less mature neonates, lower birth weight and higher percentage of women undergoing cesarean section, which in our hospital is only performed upon medical indication. These results support that this comorbidity precipitates births more prematurely and leads to the development of complications indicating hospitalization of the mother before the usual term.

Our analysis showed that the main clinical risk factor for preeclampsia was personal history of preeclampsia in a previous pregnancy, with an OR of 9.21 (95% confidence interval: 3.22–28.26), but this obviously only applies to the subset of patients in our study that were not primigravidas. Another limitation of this analysis is that we have excluded other clinical factors that are known to predispose patients to preeclampsia, such as multiple gestation, chronic hypertension, autoimmune diseases, etc. Age was detected as a risk factor, however minor, with an OR of only 1.04, possibly due to a bias introduced by enrolling slightly older women in the preeclampsia group, despite not reaching statistical significance.

Among the genetic risk factors, it was found that only the SNPs affecting eNOS were significantly associated with preeclampsia. These data agree with those from a recent meta-analysis reporting on European populations in which this association was found[41]. We did not detect an association with SNPs in genes encoding MMP-2 and MMP-9. While this confirms previous observations[30,36,38,42], the change in MMP expression already observed by other studies in association with these SNPs may still be relevant[29,37]. MMPs may be more important in the process of invasion of the spiral arteries, which is performed by trophoblasts. Since trophoblasts carry fetal DNA, such SNPs may be more important if present in the fetal DNA rather than in maternal DNA, and new studies should be designed to address this possibility. Even though no particular genotype for the VNTR in intron 4 was associated with preeclampsia in our population, we found a great variety of alleles in our population, with the appearance of the rare allele 6, and a higher frequency of the allele with 4 repetitions amongst controls. It has been recognized that Brazil has a population with wide genetic admixture[43,44] and the presence of these alleles could be explained by this multiethnic background.

In multivariate analysis, the only SNP that remained statistically significant for the risk of preeclampsia after correction for age, height and weight, and that was independent from other polymorphisms, was *NOS3* T-786C, with an OR 3.56 (95% CI: 1:23 to 10:31). The failure to identify the association of the G894T SNP with preeclampsia reported in other studies may derive from differences in sample size, more mixed genetic background, or different inclusion criteria. We have chosen to include all preeclamptic patients regardless of severity, while some studies have used only the most severe spectrum of the disease with blood pressure over 160/100mmHg. A study in a Brazilian population found an association of G894T with preeclampsia occurring after 34 weeks of gestation, but haplotype analyses on time of onset and occurrence of preeclampsia both included the -786C allele[45]. The same group found no association between individual SNPs in the *NOS3* gene with the occurrence of preeclampsia[46], and no differences in nitrite levels across different *NOS3* genotypes in preeclamptic women[47]. These discrepancies within the same region of Brazil may nonetheless reflect some ethnic differences between the populations studied and, differently from reported by one of the other studies[47], we have included patients with HELLP syndrome, and our preeclamptic group did not differ significantly in BMI from the control population, which may have previously influenced the power to detect the association between preeclampsia and this SNP. It has been demonstrated that *NOS3* T-786C polymorphism reduces eNOS mRNA expression in the placenta[48]. Therefore, this SNP may result in enzyme deficiency with reduced bioavailability of NO in the placental tissue, possibly affecting the placentation, trophoblastic invasion of the spiral arteries, and causing ischemia by local vasoconstriction. Thus, placental circulation could be primarily predisposed to an imbalance in favor of a constant reduction of placental blood flow, which can explain the development of placental insufficiency, an important pathophysiological mechanism in the development of preeclampsia. Limitations of this study include not having performed measurement of NO or its metabolites in blood samples to correlate with genotypes and strengthen this hypothesis.

Our data also agree with *NOS3* polymorphism being associated with more severe forms of preeclampsia[49], and in the present study, this SNP was particularly associated with the most severe complications (HELLP syndrome and eclampsia). Taken together, our genetic analysis and previous evidence in the scientific literature support that this SNP may not only be associated, but actually be implicated[50] in the pathogenesis of preeclampsia. NO deficiency can also exacerbate platelet activation, which favors the formation of microthrombi, generating microangiopathic hemolytic anemia and thrombocytopenia, typically found in HELLP syndrome. Intravascular hemolysis results in free hemoglobin that readily reacts with available NO, consuming even more of the already low levels of NO, a vicious circle with decreasing NO bioavailability. Therefore, the association of the *NOS3* polymorphism with preeclampsia and the occurrence of HELLP and eclampsia indicate an important role of NO in the pathophysiology of this disease. Therapeutic strategies that improve NO bioavailability may be useful approaches to be investigated as novel treatment of severe forms and as preventative measure of complications of preeclampsia.

In summary, our data support that *NOS3* T-786C polymorphism is the most important and independent genetic factor in this study associated with the occurrence of preeclampsia in a Brazilian population, and may predispose patients to more severe complications, such as HELLP syndrome and eclampsia.
